# Bacterial accumulation in intestinal folds induced by physical and biological factors

**DOI:** 10.1186/s12915-024-01874-5

**Published:** 2024-04-05

**Authors:** Jinyou Yang, Toma Isaka, Kenji Kikuchi, Keiko Numayama-Tsuruta, Takuji Ishikawa

**Affiliations:** 1 School of Intelligent Medicine, China Medical University, Shenyang, 110122 China; 2 Department of Biomedical Engineering, Graduate School of Biomedical Engineering, Tohoku University, 6-6-01 Aoba, Sendai, 980-8579 Japan; 3 Department of Finemechanics, Graduate School of Engineering, Tohoku University, 6-6-01 Aoba, Sendai, 980-8579 Japan

**Keywords:** Gut microbiota, Bacterial accumulation, Zebrafish intestine, *Escherichia coli*, Mathematical analysis

## Abstract

**Background:**

The gut microbiota, vital for host health, influences metabolism, immune function, and development. Understanding the dynamic processes of bacterial accumulation within the gut is crucial, as it is closely related to immune responses, antibiotic resistance, and colorectal cancer. We investigated *Escherichia coli* behavior and distribution in zebrafish larval intestines, focusing on the gut microenvironment.

**Results:**

We discovered that *E. coli* spread was considerably suppressed within the intestinal folds, leading to a strong physical accumulation in the folds. Moreover, a higher concentration of *E. coli* on the dorsal side than on the ventral side was observed. Our in vitro microfluidic experiments and theoretical analysis revealed that the overall distribution of *E. coli* in the intestines was established by a combination of physical factor and bacterial taxis.

**Conclusions:**

Our findings provide valuable insight into how the intestinal microenvironment affects bacterial motility and accumulation, enhancing our understanding of the behavioral and ecological dynamics of the intestinal microbiota.

**Supplementary Information:**

The online version contains supplementary material available at 10.1186/s12915-024-01874-5.

## Background

The gut microbiota plays a vital role in maintaining host health by affecting metabolism, immune function, and development [1]. This microbial community has co-evolved with its host over time to provide benefits, such as digestion, production of nutrients, detoxification, protection from pathogens, and immune regulation [2–4]. The accumulation of bacteria and their spatial distribution within the gut are dynamic processes influenced by factors, such as the gut microenvironment, bacterial motility, host-microbe interactions, and ecological competition among bacterial species [5–8]. Bacterial accumulation near the intestinal wall is particularly important because it can lead to the formation of a biofilm. Gut microbial biofilms protect the bacterial population from host immune responses and antibiotics [9] and are closely related to colorectal cancer [10, 11]. Understanding the behavior and accumulation of bacteria within the gut is of great importance, as it provides insight into the complex interactions between the microbiota and the host.

Zebrafish (*Danio rerio*) is a highly favored model organism in gut microbiota research due to its small size, high fecundity, early optical transparency, rapid external development, and good laboratory husbandry [12–14]. The optically transparent nature of the zebrafish larvae allows for high-resolution in vivo imaging of gut physiology and microbial dynamics during larval development [13, 15–19]. The intestinal environment of the zebrafish affects the motility of bacterial species, including *Escherichia coli* (*E*. *coli*), which has lower motility in the digestive tract of zebrafish [20]. Studies by Parthasarathy et al. using the zebrafish model have shown that bacteria are not uniformly distributed throughout the intestines, but exhibit strong localization preferences [18, 21–23]. The composition and distribution of the microbial communities in the zebrafish gut are affected by biological and physical factors, such as host-mediated spatial structure, bacterial taxis, gut nervous system effects, and competition with microbial populations [18, 21–24]. However, bacterial accumulation near the intestinal wall has not been quantified in detail, and the mechanism of accumulation has not been fully elucidated.

The physical effects of bacterial aggregation near a wall have been investigated in several in vitro experiments and hypotheses. Local concentrations of bacteria near flat walls can exceed bulk concentrations by several times or more, which can be explained by hydrodynamic and steric effects [25–27]. Bacterial accumulation is strongly affected by wall geometry. Bacterial residence time near the wall is reduced by the surface curvature [28] and bumps [29]. Bacterial adhesion can also be reduced by surface roughness [30], submicrometer crevices [31], and a nanoporous surface [32]. However, there are no direct measurements of how bacterial accumulation is affected by the actual fold structure of the intestinal wall in vivo. Therefore, the importance of physical factors compared to biological factors in the distribution of bacteria in the gut is unknown.

In this study, we investigated the behavior and accumulation of *E*. *coli* in the intestine of larval zebrafish, with a specific focus on the influence of the gut microenvironment. Our results show that *E*. *coli* exhibited different motility patterns with changes in the intestinal microenvironment, such as viscosity and wall geometry. Notably, we observed a greater accumulation of *E*. *coli* within the intestinal folds and confirmed through in vitro microfluidic experiments that the geometry of the intestinal wall favored the accumulation of *E*. *coli* within the folds. Furthermore, we observed a higher density of *E*. *coli* on the dorsal side of the intestines, suggesting a biological mechanism. Our theoretical analysis revealed that the overall distribution of *E*. *coli* in the intestine is established by a combination of physical factor and bacterial taxis. These findings provide valuable insight into the effects of the intestinal microenvironment on bacterial motility and accumulation. The results contribute to a deeper understanding of the behavior and ecological dynamics of the intestinal microbiota and have important implications for the study of health and disease.

## Results

### Bacterial behavior in the zebrafish larval intestine

#### Geometric characteristics of the intestinal folds

**Fig. 1:**
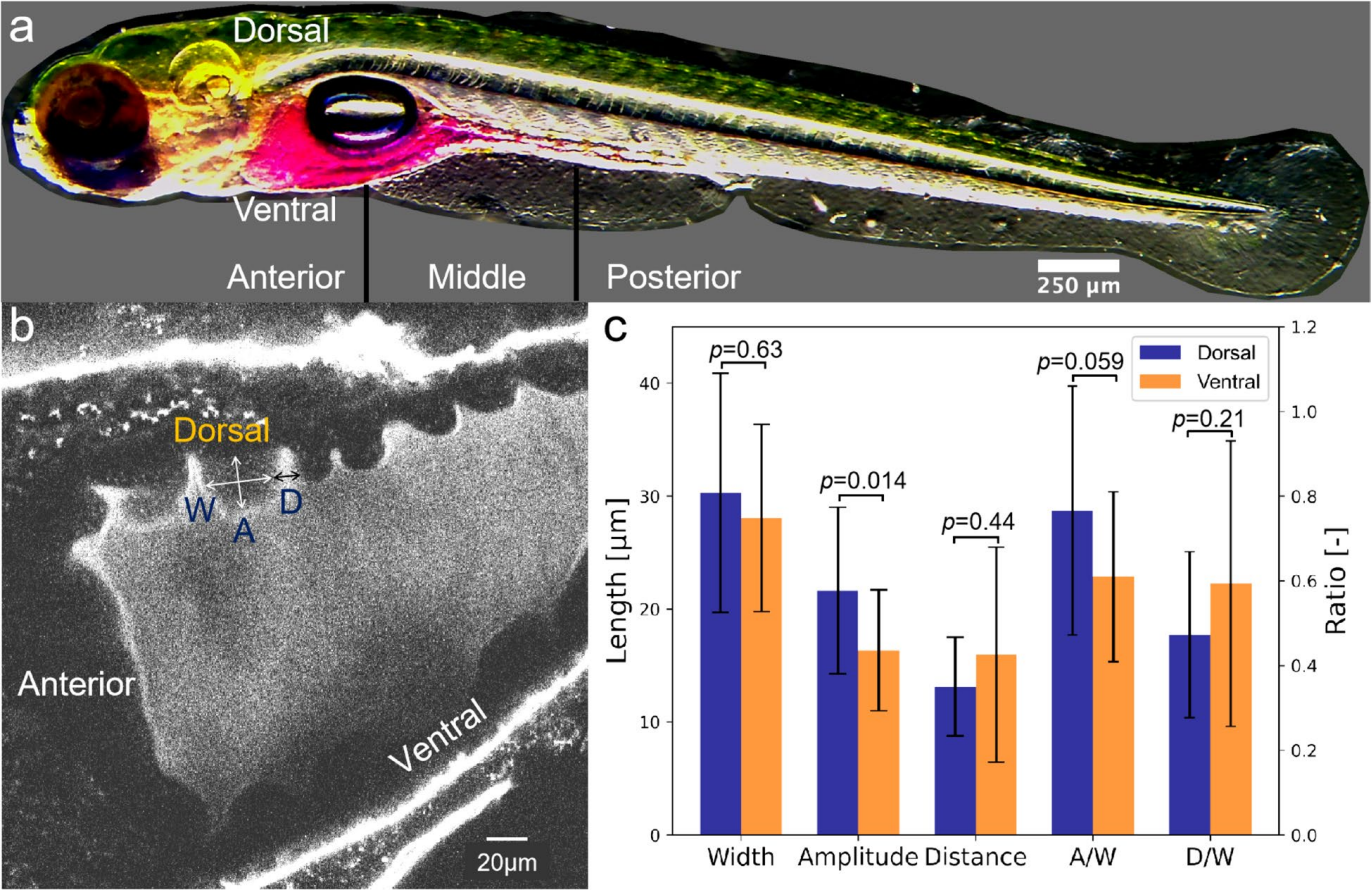
Structural features of the anterior intestinal wall fold 7 days post-fertilization (dpf). **a** Larval zebrafish at 7 dpf. The intestine was highlighted for illustration by orally gavaging with Rhodamine B dye. The intestine was divided into three different segments: anterior, middle, and posterior. **b** Definition of the amplitude *A*, width *W*, and distance between the folds in the intestinal wall *D*. **c** Geometric features of the intestinal folds on the dorsal and ventral sides. *A*/*W* is the ratio of amplitude to width, and *D*/*W* is the ratio of distance to width. *p* values as determined by the Mann–Whitney–Wilcoxon test, and error bars indicate standard deviation (*N* = 6 fish)

First, we investigated the geometric characteristics of the larval zebrafish intestine. We visualized the intestine of zebrafish 7 days post-fertilization (dpf), from the anterior intestine to the anus 2 h after injecting 10 mM rhodamine B solution. As shown in Fig. 1a, the intestine was divided into three segments, the anterior intestine, middle intestine, and posterior intestine. The cross-section perpendicular to the centerline of the anterior intestine is larger than the other parts. Figure 1b is a confocal fluorescence microscopic image at the central cross-section parallel to the centerline of the anterior intestinal lumen. The intestine was dyed by feeding PlasMem Bright Red dye. Images of other cross-sections of the anterior intestinal lumen are provided in Additional file 1: Movie S1. The images show the characteristic geometries of the anterior intestinal folds. To quantitatively assess the geometric characteristics of these folds, we measured the width, amplitude, and distance between two folds. In addition, we calculated the ratio of amplitude to width and the ratio of distance to width, as shown in Fig. 1c. The width of the folds on the dorsal and ventral sides of the anterior intestine was approximately 30 μm, the amplitude was 20 μm, and the distance between the folds was 15 μm. A small difference in wall geometry was detected between the dorsal and ventral sides.

#### Bacterial motility in the intestine

**Fig. 2:**
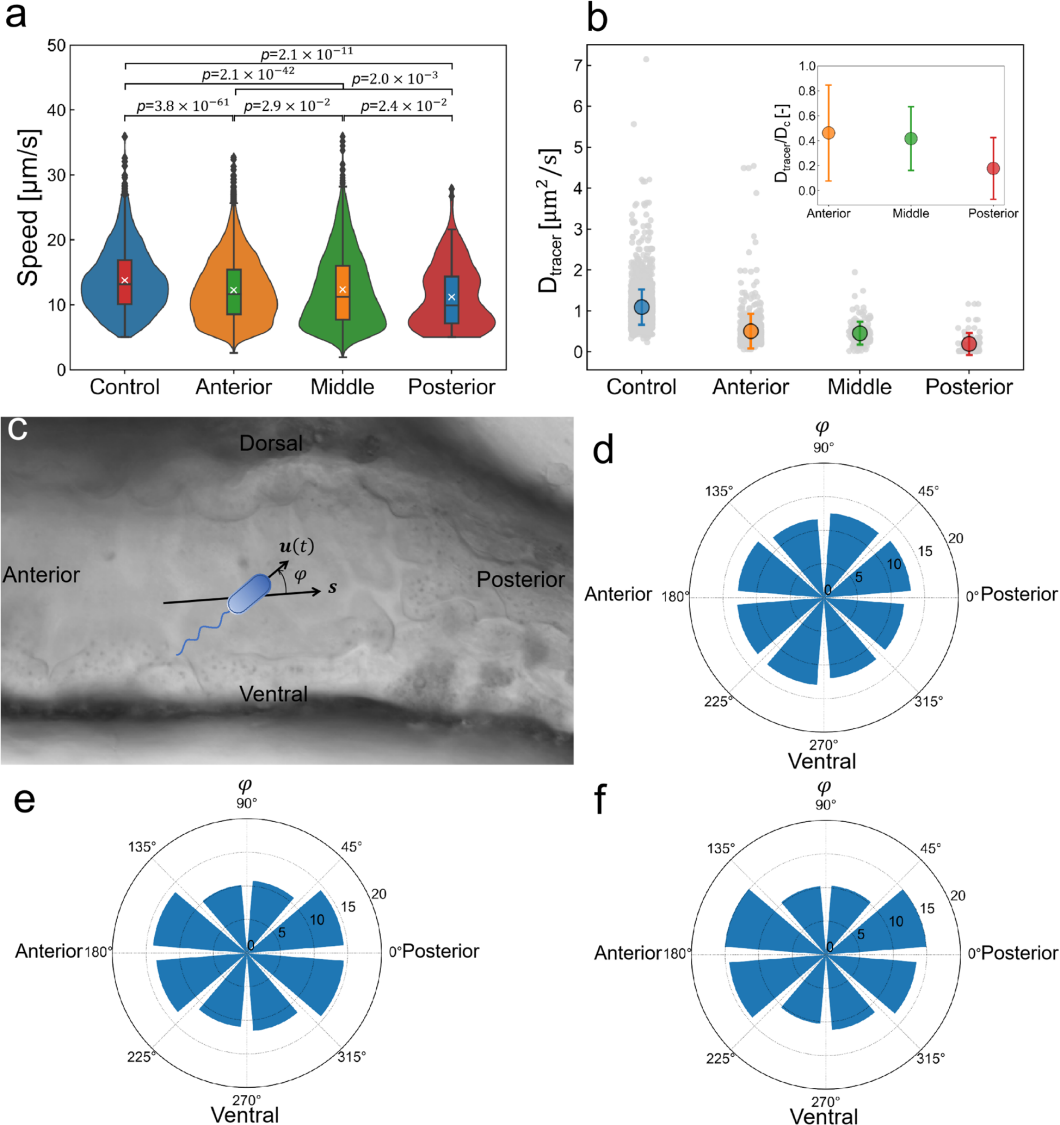
Bacterial movement and the microenvironment of the zebrafish larval intestine. **a** Swimming speeds of the bacteria in the three segments. The control indicates bacteria in culture fluid on a glass slide. Solid symbols and error bars indicate the mean and standard deviation, respectively (*N* = 6 fish). *p* values are determined by the Mann–Whitney–Wilcoxon test. **b** Diffusion coefficients of fluorescent tracer particles in the three segments. All data points are gray. The inset indicates the ratio of the diffusion coefficients in the three segments to the control. **c** Bacterial orientation *φ* was defined as the angle from the centerline vector ***s*** of the intestine. **d**–**f** Probability density distribution of the bacterial orientation in the three segments after cell distribution reached a steady state: **d** anterior intestine, **e** middle intestine, and **f** posterior intestine

Figure 2a shows the swimming speed of the bacteria in the different intestinal segments. A dilute suspension of *E*. *coli* was injected into the anterior intestine with a microneedle using the microgavage method. *E*. *coli* trajectories were recorded by a high-speed camera using a 40× oil objective lens. Peristalsis of the intestine was intermittent, and the trajectories were recorded under conditions where peristalsis ceased and there was little background flow. The control was a bacterial solution placed on a glass slide. The bacteria in the zebrafish larval intestine were significantly slower than those in the control. The bacteria moved progressively slower from the anterior intestine to the posterior intestine.

To understand this phenomenon, we measured the diffusion coefficient of 0.5-μm fluorescent particles in each intestinal segment, so that local viscosity was estimated from the Stokes-Einstein equation. To characterize the viscosity of the intestine in the microenvironment where *E*. *coli* was present, we injected fluorescent particles and an *E*. *coli* solution into the intestine at a 1:1 ratio. The diffusion coefficient was calculated from the mean squared displacement of the particles, as explained in the “Methods” section. Figure 2b shows the variation in the diffusion coefficient within the zebrafish larval intestine. The inset in Fig. 2b indicates the ratio of the diffusion coefficients at different segments to the control. The diffusion coefficient in the anterior intestine was approximately half of that in the control, indicating that the viscosity in the anterior intestine was about twice as high as that in the control. Similar results were reported by Taormina et al. [33]. The diffusivity decreased from the anterior to the posterior intestine, i.e., viscosity increased from the anterior to the posterior intestine. Since the higher viscosity lowers the swimming speed, the differences in the local viscosity of the intestine may be partly responsible for the decrease in swimming velocity toward the posterior intestine.

*E*. *coli* swimming direction was also affected by the microenvironment in each intestinal segment. The orientation angle φ of swimming velocity *u*(t) relative to the centerline was defined as shown in Fig. 2c. Figure 2d–f shows the probability distribution of the orientation in each intestinal segment after cell distribution reached a steady state. The bacteria exhibited isotropic orientation in the anterior intestine, with almost uniform probability distribution in all directions (Fig. 2d). By contrast, the bacteria oriented more along the centerline in the middle and posterior intestines (Fig. 2e, f). The effect of the wall boundary was more significant in the middle and posterior intestines, which led to the directional movement of *E*. *coli*.

### The folds facilitate bacterial accumulation

#### Bacterial accumulation in the zebrafish larval intestinal folds

**Fig. 3:**
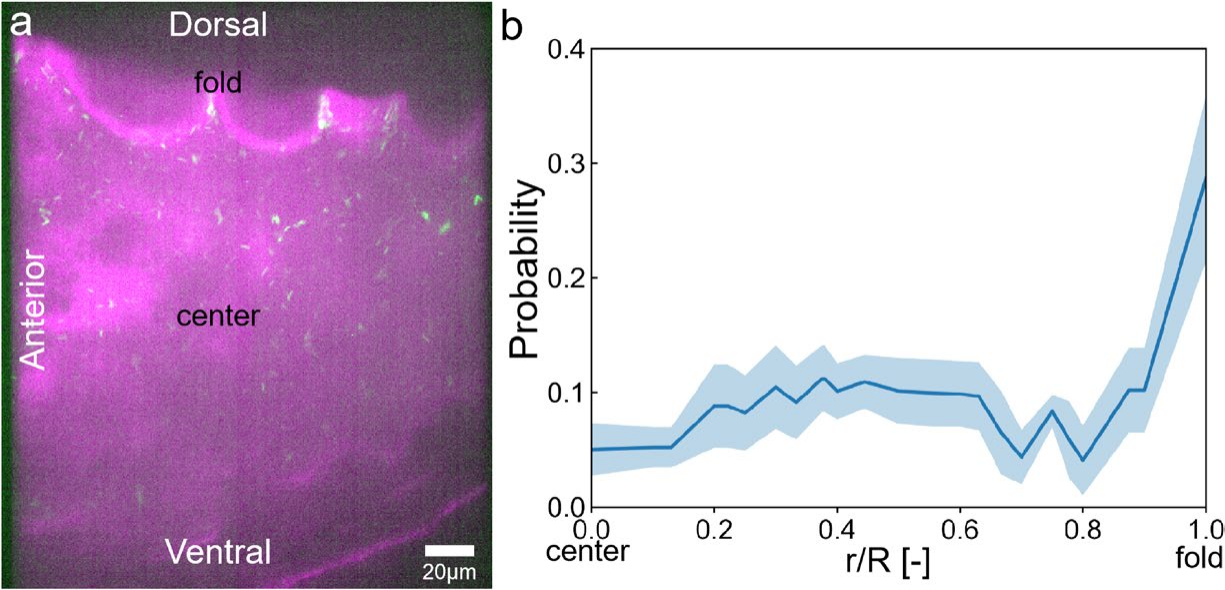
Bacterial distribution in the anterior intestine. **a** Confocal fluorescence microscopic field-of-view image. Magenta indicates the intestinal wall and green indicates the bacteria. **b** Bacterial probability density distribution varies with position from the intestinal center to the fold; *r* is the coordinate from the center to the wall, and *R* is the position at the bottom of the folds; the blue line is the mean, and the shaded regions mark the standard deviation (*N* = 3 fish)

*E*. *coli* tended to accumulate in the folds of the anterior intestinal wall, as shown in Fig. 3a and Additional file 1: Movie S2. Figure 3b shows the probability density distribution of bacteria from the center of the anterior intestine to the intestinal wall folds. The results show that the intestinal folds accumulated about five times more bacteria than the bulk. In contrast, the fluorescent tracer particles showed a normal distribution with more in the center and less near the folds (Additional file 2: Fig. S4).

**Fig. 4:**
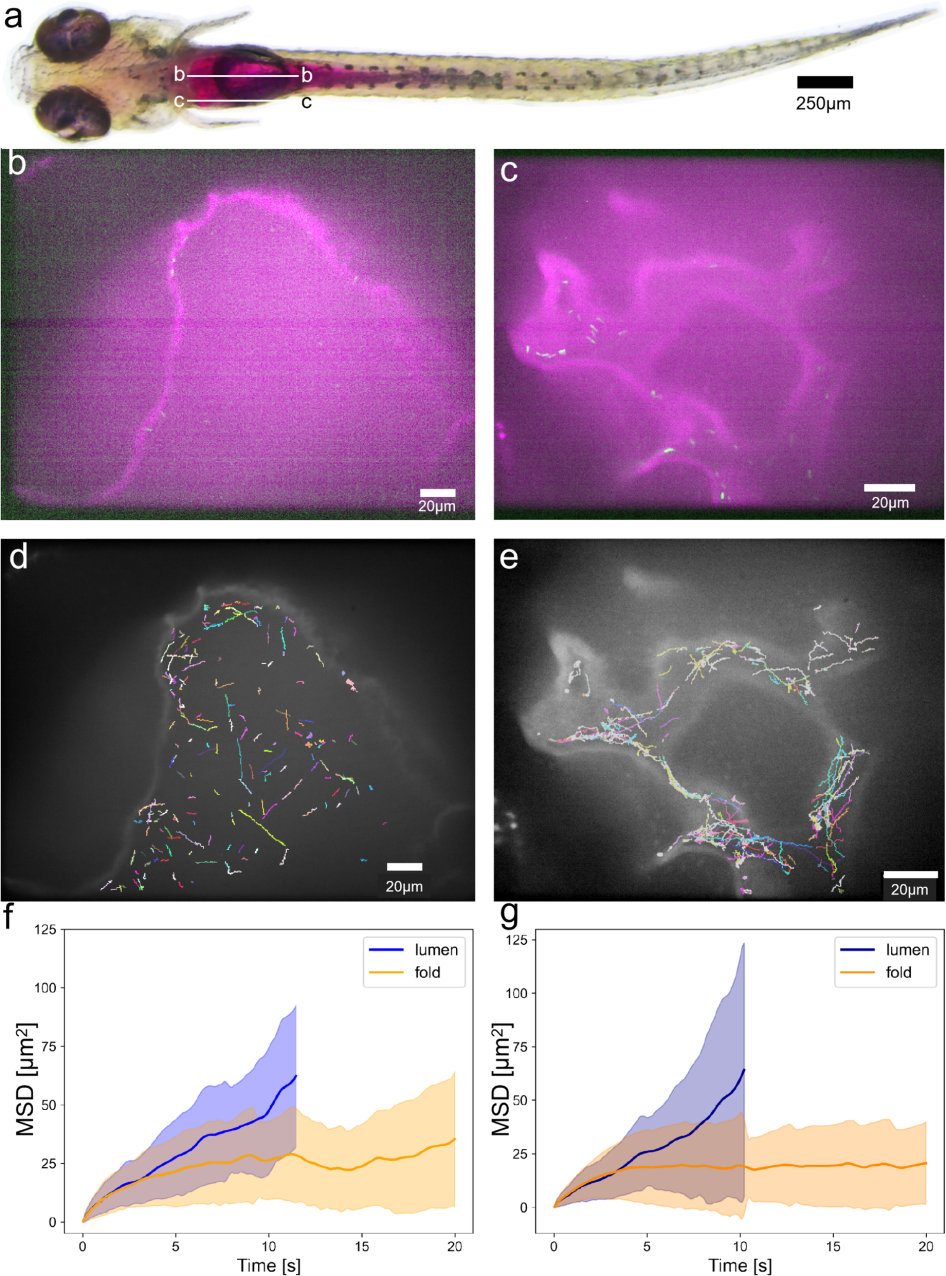
Difference in bacterial behavior between the lumen and the folds. **a** Location of the lumen and folds on the abdomen of supine zebrafish. **b**, **c** Distribution of bacteria in the confocal fluorescence microscopic field of view in the middle cross-section of the anterior intestinal lumen (**b**) and intestinal wall folds (**c**). **d** and **e** represent the trajectories of the swimming bacteria in **b** and **c**, respectively. **b**–**e** The results 0 h after injection. **f**, **g** The mean squared displacement (MSD) in the anterior intestinal lumen or folds varied at 0 h (*N* = 8 fish) (**f**) and 1 h (**g**) after the injection. The lines represent the mean, and the shaded regions indicate the standard deviations (*N* = 6 fish)

The behavior of the bacteria in the intestinal folds was further investigated to elucidate the mechanism of bacterial accumulation near the wall. Figure 4b and c show the confocal fluorescence images of bacteria in the central cross-section of the anterior intestine and in the folds of the anterior intestinal wall, respectively. Figure 4d and e represent the trajectories of swimming bacteria in Fig. 4b and c. Additional results are provided in Additional file 2: Fig. S1 and Fig. S2. Many of the trajectories of bacteria within the intestinal folds were longer than those in the central cross-section, despite the same observation time, suggesting that bacteria in the central cross-section quickly left the focal plane of observation, while bacteria in the folds did not.

We also analyzed the mean squared displacement (MSD) of bacteria in the central cross-section and the folds just after injecting the bacteria (Fig. 4f) and 1 h after injection (Fig. 4g). The MSD in the central cross-section increased almost linearly with time, indicating that the spread of bacteria was diffusive. The MSD in the folds did not increase significantly in the long-term region. Thus, bacterial spread was suppressed by the folds, as seen in Additional file 1: Movie S3. This suppressed cell flux from the folds to the bulk resulted in a greater accumulation of *E*. *coli* within the folds than in the bulk, which was confirmed using a mathematical model. We see that MSD curves in the lumen and in the folds almost overlap for the first few seconds up to MSD ~ 20 μm^2^, i.e., a distance of about 4.5 μm. This distance is much smaller than the distance of the folds (14~17 μm) shown in Fig. 1c. Hence, the effect of the folds becomes small and the orientational change of *E. coli* shows similar tendencies in the lumen and in the fold. A comparison of Fig. 4f (0 h) and g (1 h) indicates that bacterial motility was almost unchanged during the hour, and the suppressed spread of bacteria in the folds was maintained for a long time.

#### Bacterial accumulation in the folds of an in vitro microchannel

**Fig. 5:**
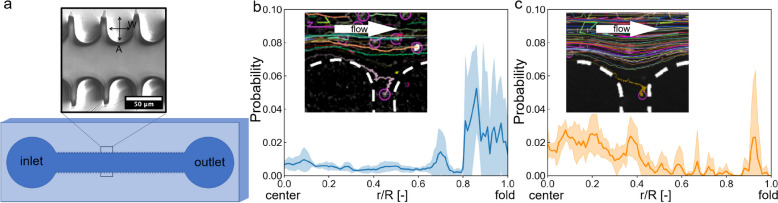
In vitro bacterial behavioral experiments using a microchannel with folds. **a** Schema of the microfluidics device with many folds. A suspension of *E*. *coli* flows from the inlet to the outlet. **b**, **c** Probability density distribution varied with the position in the microchannel from the center to the fold, and the inset indicates the trajectory of the flow; *r* is the coordinate from the center to the wall, and *R* is the position at the bottom of the folds: **b** *E*. *coli*, **c** particles. The line represents the mean, and the shaded regions indicate standard deviations (*N* = 5)

Next, a microfluidic approach was used to investigate whether the accumulation of bacteria in the folds was due to the geometry of the intestine. As shown in Fig. 5a, we fabricated a polydimethylsiloxane (PDMS) microfluidic device using a soft photolithography technique. The wall geometry of the device was designed to mimic the folds in the anterior intestine. The background flow was applied from the inlet to the outlet with a flow rate of 0.01 µL/min, which mimicked the background peristaltic flow in the intestine. The probability density distribution of bacteria and fluorescent particles in the microfluidic channel is shown in Fig. 5b and c, respectively. The insets to the figures indicate the trajectories of the bacteria or fluorescent particles near the folds. The bacteria exhibited a higher probability density near the microchannel folds, indicating that the bacteria physically accumulated in response to fold geometry. A comparison of Fig. 5b and c illustrates the importance of cell motility in bacterial accumulation in the folds. These results are consistent with our in vivo observation, in which motile bacteria are accumulated in the folds while tracer particles are not.

### Spatially asymmetric distribution of bacteria in the zebrafish larval intestine

#### Stronger accumulation of bacteria on the dorsal side of the intestine

**Fig. 6:**
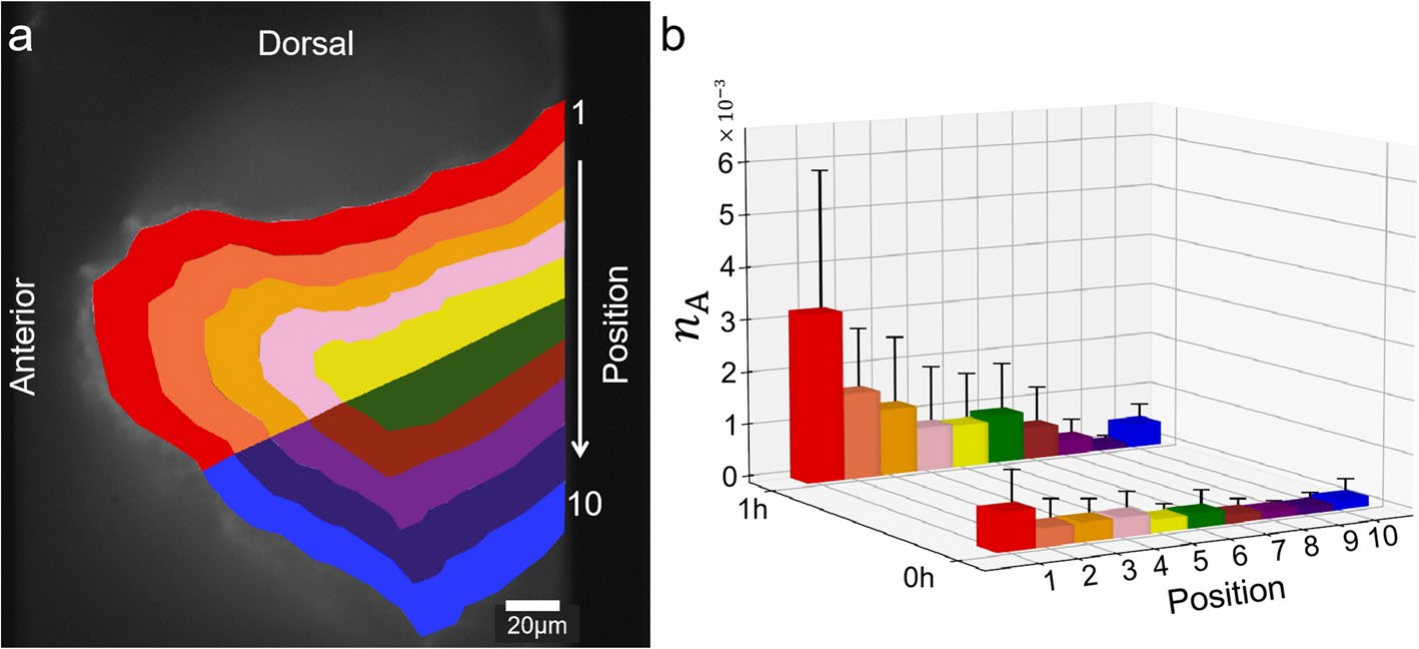
Asymmetric distribution of bacteria between the dorsal and ventral sides. **a** Definition of regions 1–10. **b** Distribution of areal density of bacteria from regions 1 to 10 at 0 and 1 h after injection (*N* = 6 fish)

A spatially asymmetric distribution of bacteria was observed in the intestinal lumen. Ten regions were defined to further explore this distribution. The regions were separated symmetrically from the dorsal to the ventral side, as shown in Fig. 6a. Figure 6b represents the areal number density of bacteria from regions 1 to 10 in the anterior intestine at 0 and 1 h after injection. The results revealed a significantly higher density of bacteria on the dorsal side than on the ventral side. The density tended to increase from the ventral to the dorsal sides throughout the bulk, with a local increase near the intestinal wall. A similar asymmetric distribution of bacteria and local accumulation near the wall were observed in the middle intestine (Additional file 2: Fig. S3). An increase in the density of bacteria from 0 to 1 h after injection was seen, although the distribution itself looked similar. In contrast, the fluorescent tracer particles showed a normal distribution with more in the middle and less on the sides (Additional file 2: Fig. S4).

#### Bacterial distribution induced by combined physical and biological factors

We have shown that bacteria accumulated in the folds by the physical mechanism of wall geometry (cf. Fig. 5b), but this mechanism does not explain the asymmetric distribution between the dorsal and ventral sides, where there is no significant difference in wall geometry (cf. Fig. 1c). Given the dorsal to ventral gradient of the bacterial distribution in bulk, we inferred that there are unknown biological factors in the bulk that caused directional movement of cells toward the dorsal side, i.e., taxis. To test this hypothesis, we constructed a continuum model that accounted for the physical factor of a decrease in cell flux from the wall to the bulk and the bacterial taxis from the ventral to the dorsal side. The details of the continuum model are explained in the “Methods” section and the Additional file 3.

**Fig. 7:**
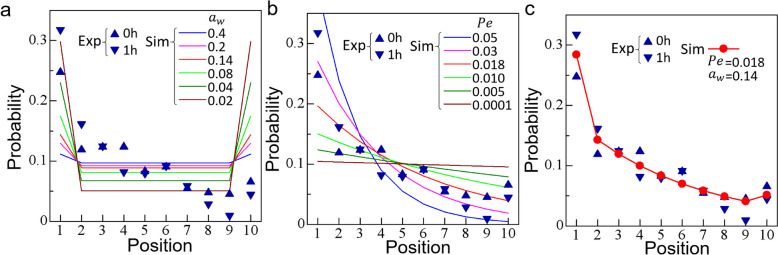
Comparison of the probability distribution of the areal density of bacteria between the experiment and the simulation. Blue triangles indicate experimental results of 0 and 1 h. Colored lines and red circles indicate the numerical results. **a** Effect of ${\alpha }_{w}$ on the accumulation of cells near the wall, where ${\alpha }_{w}$ is a dimension-free parameter indicating the ratio of ensemble-averaged bacterial velocity away from the wall to that toward the wall. **b** Effect of ${\text{Pe}}$ on the bulk gradient cell distribution. **c** The least-square fit of the numerical results with the experimental results (${\text{Pe}}=0.018, {\alpha }_{w}=0.14, {r}^{2}=0.92$)

The results of Fig. 6b were converted to a probability density distribution of bacteria and are replotted in Fig. 7, in which the distribution is almost the same as during the 1 h. We first analyzed the bacterial distribution caused only by the physical factor of wall accumulation, in the absence of the bacterial taxis. Figure 7a shows the effect of the wall accumulation parameter ${\alpha }_{w}$ indicating the ratio of ensemble average bacterial velocity away from the wall to that toward the wall. Wall accumulation was reproduced by this model, but the bacterial distribution became symmetric between the dorsal and ventral sides. This was different from the experimental results: physical entrapment in the folds alone did not explain the experimental results. Figure 7b shows the bacterial probability density induced by the bacterial taxis. The Péclet number (Pe) indicates the effect of directional movement relative to diffusion. The bulk density gradient was reproduced, but the wall accumulation at position 10 was not. Thus, bacterial taxis alone do not explain the experimental results. Finally, the combination of physical factor and bacterial taxis on the bacterial distribution yielded the results shown in Fig. 7c. The bulk gradient and wall accumulation were reproduced. These results illustrate that the distribution of *E*. *coli* in the zebrafish larval intestine was established by a combination of physical factor and bacterial taxis.

## Discussion

We conducted a comprehensive quantitative analysis of the microenvironment in the 7-dpf zebrafish intestine, focusing on the structure of the intestinal folds and viscosity. The results revealed distinct variations in the microenvironment anterior to posterior in the intestine, resulting in different bacterial motility patterns. The anterior intestinal space was characterized by larger dimensions and lower viscosity, facilitating faster and isotropic movement of bacteria. By contrast, the middle and posterior intestines exhibited narrower dimensions and higher viscosity, leading to slower bacterial motion along the longitudinal axis of the intestine.

Bacterial accumulation near a wall has been observed in many in vitro biophysical experiments [26–28, 31, 32, 34]. Similar to these studies, swimming bacteria in the gut were attracted to the intestinal wall in vivo. This suggests that the findings obtained by biophysics contribute to studies of the intestinal flora. The anterior intestinal wall of zebrafish has folds with a width of about 30 μm, amplitude of 20 μm, and distance of 15 μm. Notably, the movement of bacteria was restricted upon entering such intestinal folds, resulting in an increased accumulation of bacteria within these folds. We rigorously confirmed that this accumulation phenomenon was primarily driven by the physical geometry of the intestinal fold, as evidenced by the in vitro microfluidic experiments.

Furthermore, we observed an asymmetric spatial distribution of bacteria in the zebrafish intestine. Specifically, bacterial density was significantly higher on the dorsal side than the ventral side, and this asymmetry persisted over time even as the overall bacterial density increased. Importantly, converting the bacterial density distribution into a probability distribution revealed that this asymmetric spatial pattern remained relatively stable without significant changes over time. A theoretical analysis further indicated that this phenomenon was attributed to a combination of the physical geometry of the intestinal folds and the bacterial taxis. This conclusion suggests that both biology and physics are important in understanding gut flora.

## Conclusions

By providing precise quantitative insight into the microenvironment of the zebrafish intestine and elucidating the behavioral and spatial dynamics of bacteria, our study enhances the understanding of the intricate behavior of bacteria affected by physical factor and bacterial taxis in the gut microenvironment. These findings have important implications for advancing our knowledge of the role of gut microbiota in health and disease.

## Methods

### Zebrafish handling and preparation

All zebrafish breeding and handling were as described in our previous study [35]. Wild-type zebrafish provided by the National BioResource Project were bred in water tanks maintained at 28.5 °C and a 14 h:10 h light to dark cycle. The adult male and female zebrafish were bred separately in plastic cases within the same water tank to control the spawning cycle. A pair of male and female zebrafish were placed in a small box separated by baffles before the dark cycle in preparation for spawning. The baffles were removed to allow the fish to spawn the next morning. The fertilized embryos were collected 1 h after natural spawning, washed with Milli-Q water (ultra-pure water), and placed in E3 water (5 mM NaCl, 0.17 mM KCl, 0.33 mM CaCl_2_, and 0.33 mM MgSO_4_) at 28.5 °C for incubation. The larvae were sustained on yolk-derived nutrients and were not fed until 7 dpf for the experiments. PlasMem Bright Red dye (2 µL; Dojindo Laboratories, Kumamoto, Japan) was added to 5 mL of E3 water to label the intestinal structure, and the fish were placed in the water for 4 h before the experiment.

### Bacterial strains and culture

*E*. *coli* strain MG1655 expressing AcGFP transformed by pAcGFP1 (Clontech TaKaRa, Shiga, Japan) was used in our experiments. Frozen stocks of the bacteria were maintained in 50% glycerol at − 80 °C. A 100-µL aliquot of *E*. *coli* frozen stock was added to 10 mL of tryptone broth supplemented with 10 µL of 100 mg/mL ampicillin sodium (FUJIFILM Wako, Tokyo, Japan) and 200 µL of 100 mM isopropyl β-D-1-thiogalactopyranoside (IPTG) (FUJIFILM Wako) and incubated overnight at 33 °C. Ampicillin was used to select transformed *E*. *coli*, and IPTG was added to induce the expression of AcGFP. Approximately 100 µL of the culture was resuspended in 10 mL of the same medium and incubated at 33 °C with shaking at 200 rpm for 8 h, until the OD_600_ reached approximately 0.5.

### Microgavage of zebrafish larvae

The 7-dpf larval zebrafish were mounted for imaging as described in our previous study[35]. The larval zebrafish were placed on a 3% agarose gel bed and the posture was adjusted to lie flat on the gel bed. Agarose powder (0.9 g) and water (30 mL) were stirred in a flask and dissolved by boiling. The agarose solution was poured into a bed mold cut from rubber and cooled at room temperature. Larval zebrafish were immersed in 3% methylcellulose solution, which is non-toxic and highly viscous (methylcellulose no. 1500; Nacalai Tesque, Tokyo, Japan), to secure it on the gel bed. A 100-mL aliquot of 3% methylcellulose was prepared by freezing 65 mL of water at − 20 °C for 30 min, heating 35 mL of water to 80 °C in a glass beaker, adding 6 g of methylcellulose, and stirring until all particles were wetted and uniformly dispersed. Ice-cold water was added, mixed, and cooled at 4 °C.

A hydraulic microinjector (Nanoject III, Drummond Scientific Co., Broomall, PA, USA) was used for microgavage. A tapered glass capillary filled with olive oil (Nichi-iko, Tokyo, Japan) was installed on the tip of the injector. The tapered glass capillary was fabricated by pulling a glass capillary (3-000-203-G/X, Drummond Scientific) with the PC-100 puller device (Narishige, Kyoto, Japan). Then, the capillary needle was cut to adjust the edge diameter to 30 µm using the Micro Forge MF2 (Narishige).

A glass needle filled with sample material was inserted through the mouth under a stereomicroscope and used to inject the solution into the anterior intestine. The zebrafish larvae were anesthetized for all procedures in 120 µg/mL tricaine solution (ethyl 3-aminobenzoate methanesulfonate suspended in ultra-pure water; Sigma-Aldrich, St. Louis, MO, USA).

### Microfluidic device

The PDMS microfluidic device was fabricated using a conventional soft photolithography technique. As shown in Fig. 5a, the device consisted of an inlet, an outlet, and a central chamber with folds like the zebrafish larval anterior intestinal folds (Fig. 1b). The height, length, maximum width, and minimum width of the microchannel are 100 μm, 14 mm, 128 μm, and 35 μm, respectively. The fold amplitude is 47 μm, and the width is 39 μm. The PDMS device was bonded to glass coverslips using a plasma cleaning process in which they were placed inside a plasma cleaner (PIB-20 vacuum device) for 2 min, bonded, and placed on a 65°C hot plate set for 30 min for optimal bonding. A high-precision syringe pump (PHD ULTRA 70-3007, Harvard Apparatus, Holliston, MA, USA) was used to introduce the bacterial suspension into the microchannel at a well-controlled flow rate.

### Microscopy and cell tracking

An inverted confocal fluorescent microscope (Olympus IX71, Japan) with an oil magnification objective (40 $\times$) was used to observe the swimming of the bacteria in the intestine of the larval zebrafish. A 28.5 °C thermoplate (Tokaihit, Japan) was used instead of the object stage to maintain the same conditions as the fish tanks. Videos were taken with a high-speed camera (CSD-4S, Metek, Tannersville, NY, USA) at a frame rate of 50 fps. The images were evaluated using microparticle tracking velocimetry [35, 36] and the TrackMate plug-in (Fiji) for ImageJ software (NIH, Bethesda, MD, USA). The position and trajectory of a selected bacterium can be obtained using successive images and this software.

Fluorescent carboxylate-modified particles (diameter = 0.5 µm; Ex = 580 nm; Em = 605 nm; 1:2000 diluted in ultra-pure water; Thermo Fisher Scientific, Waltham, MA, USA) were co-injected with an *E*. *coli* suspension to assess the viscosity of the zebrafish larval intestine.

### Data analysis

Bacterial trajectories were smoothed by running averages over five points. The speed of the bacteria was measured as a scalar quantity representing the distance moved between consecutive frames, divided by the time elapsed. Given the trajectory of a cell, $r\left(t\right)=\left[x\left(t\right),y\left(t\right)\right]$, where $x\left(t\right)$ is the *x*-coordinate of the cell and $y\left(t\right)$ is the *y*-coordinate of the cell; the velocity is defined by $\mathbf{v}\left(t\right)=\frac{r\left(t+\delta t\right)-{\varvec{r}}\left(t\right)}{\delta t}=v(t)\left[{\text{cos}}\varphi \left(t\right),{\text{sin}}\varphi \left(t\right)\right]$, where $\delta t$ is the time interval between two consecutive frames, speed $v(t)=\left|\mathbf{v}\left(t\right)\right|$, and $\varphi \left(t\right)\in (\mathrm{0,2}\pi )$ is the moving orientation as shown in Fig. 2c.

Diffusion coefficients were calculated from the particle trajectories in the zebrafish larval intestine or on the glass slide (control group) in an *E*. *coli* solution. It was defined as ${D}_{{\text{tracer}}}=\frac{1}{n}\sum_{i=0}^{n}\frac{\langle MSD\rangle }{4T}=\frac{1}{n}\sum_{i=0}^{n}\frac{{\left[{r}_{i}\left(T\right)-{r}_{i}\left(0\right)\right]}^{2}}{4T}$, where *n* is the number of tracking particles, *T* is time, ${r}_{i}$ is the location of particle *i*, ${\text{and}} MSD$ is the mean squared displacement for each trajectory of a particle.

### Theoretical analysis

We constructed a continuum model that accounts for the physical factor of a decrease in cell flux from the wall to the bulk and the bacterial taxis of directional movement of cells from the ventral to the dorsal side. The details of the continuum model are explained in Additional file 3.

In the bulk, the conservation of cells can be expressed using a control volume method, such as

$$\frac{\partial {n}_{i}}{\partial t}=v\frac{{n}_{i+1}-2{n}_{i}+{n}_{i-1}}{dx} +{v}_{a}\frac{{n}_{i+1}-{n}_{i}}{dx} ,$$

where ${n}_{i}$ is the density of cells in mesh $i$, $t$ is time, $v$ is the bacterial velocity, ${v}_{a}$ is the directional velocity toward the dorsal side, and $dx$ is the mesh size. Péclet number ${\text{Pe}}$ was defined as the ratio of ${v}_{a}$ to $v$, indicating the effect of the directional movement relative to the diffusion.${\text{Pe}}$ was expressed as

$${\text{Pe}}=\frac{{v}_{a}}{v}=\frac{{v}_{a}dx}{D} ,$$

where $D$ is diffusivity.

We non-dimensionalized the equation using $dx$ as the characteristic length scale and ${dx}^{2}/D$ as the characteristic time scale. The equation was transformed as

$$\frac{\partial {n}_{i}^{*}}{\partial {t}^{*}}=\left(1+{\text{Pe}}\right){n}_{i+1}^{*}-\left(2+{\text{Pe}}\right){n}_{i}^{*}+{n}_{i-1}^{*} ,$$

where $*$ indicates a dimensionless quantity. Using the Euler explicit method for time-marching, we have:

$${{n}_{i}^{*}}^{m+1}={{n}_{i}^{*}}^{m}+dt{\left[\left(1+{\text{Pe}}\right){n}_{i+1}^{*}-\left(2+{\text{Pe}}\right){n}_{i}^{*}+{n}_{i-1}^{*}\right]}^{m} ,$$

where $dt$ is the time step and $m$ is the step number.

We have the following equation for mesh 1 next to the dorsal wall:

$${{n}_{1}^{*}}^{m+1}={{n}_{1}^{*}}^{m}+dt{\left[\left(1+{\text{Pe}}\right){n}_{2}^{*}-{\alpha }_{w}{n}_{1}^{*}\right]}^{m} ,$$

where ${\alpha }_{w}$ is a dimension-free parameter indicating the ratio of ensemble-averaged bacterial velocity away from the wall to that toward the wall. Wall accumulation was expressed by using the ratio of the density of cells in the bulk to that near the wall [37].

For the last mesh $M$ next to the ventral wall, we have the following equation:

$${{n}_{M}^{*}}^{m+1}={{n}_{M}^{*}}^{m}+dt{\left[-\left({\alpha }_{w}+{\text{Pe}}\right){n}_{M}^{*}+{n}_{M-1}^{*}\right]}^{m} .$$

These equations were solved explicitly using sufficiently small $dt$ and $dx$ until convergence was satisfied, and the steady state solution was obtained.

### Supplementary Information

**Additional file 1:** **Movie S1.** Geometry of the anterior intestinal lumen of zebrafish larvae in different sections of the z-axis.** Movie S2.** Bacterial swimming behavior in the larval zebrafish anterior intestine from the fold to the center.** Movie S3.** Bacterial swimming behavior in the larval zebrafish anterior intestinal fold.

**Additional file 2:** **Figure S1.** Trajectories of swimming bacteria in the confocal fluorescence microscope field of view of the middle section of the anterior intestinal lumen.** Figure S2.** Trajectories of swimming bacteria in the anterior intestinal folds.** Figure S3.** Areal number density of bacteria in different locations of the lumen of the middle intestine (near to the anterior intestine) at 0h.** Figure S4.** Areal number density and trajectories of fluorescent tracer particles in the anterior intestinal lumen.

**Additional file 3.** The details of the continuum model.

## Data Availability

All data needed to evaluate the conclusions in the paper are included in this published article and its supplementary information files. Additional data related to this paper are available from the corresponding authors upon reasonable request.

## Abbreviations

*E. coli*: *Escherichia coli*
dpf: Days post-fertilization
MSD: Mean squared displacement
PDMS: Polydimethylsiloxane
Pe: Péclet number

## References

[1] Chow J, Tang H, Mazmanian SK (2011). Pathobionts of the gastrointestinal microbiota and inflammatory disease. Curr Opin Immunol.

[2] Wu H-J, Wu E (2012). The role of gut microbiota in immune homeostasis and autoimmunity. Gut Microbes.

[3] Rajilic-Stojanovic M, Figueiredo C, Smet A, Hansen R, Kupcinskas J, Rokkas T (2020). Systematic review: gastric microbiota in health and disease. Aliment Pharmacol Ther.

[4] Lazar V, Ditu L-M, Pircalabioru GG, Gheorghe I, Curutiu C, Holban AM, et al. Aspects of Gut Microbiota and Immune System Interactions in Infectious Diseases, Immunopathology, and Cancer. Front Immunol. 2018;9:1830. 10.3389/fimmu.2018.01830 PMC6104162 30158926

[5] Ley RE, Hamady M, Lozupone C, Turnbaugh P, Ramey RR, Bircher JS (2008). Evolution of mammals and their gut microbes. Science.

[6] Donaldson GP, Lee SM, Mazmanian SK (2016). Gut biogeography of the bacterial microbiota. Nat Rev Microbiol.

[7] Tropini C, Earle KA, Huang KC, Sonnenburg JL (2017). The gut microbiome: connecting spatial organization to function. Cell Host Microbe.

[8] Ishikawa T, Omori T, Kikuchi K (2020). Bacterial biomechanics—from individual behaviors to biofilm and the gut flora. APL Bioeng.

[9] Swidsinski A, Weber J, Loening-Baucke V, Hale LP, Lochs H (2005). Spatial organization and composition of the mucosal flora in patients with inflammatory bowel disease. J Clin Microbiol.

[10] Hold GL, Allen-Vercoe E (2019). Gut microbial biofilm composition and organisation holds the key to CRC. Nat Rev Gastroenterol Hepatol.

[11] Tomkovich S, Dejea CM, Winglee K, Drewes JL, Chung L, Housseau F (2019). Human colon mucosal biofilms from healthy or colon cancer hosts are carcinogenic. J Clin Invest.

[12] Xia H, Chen H, Cheng X, Yin M, Yao X, Ma J (2022). Zebrafish: an efficient vertebrate model for understanding role of gut microbiota. Mol Med.

[13] Flores EM, Nguyen AT, Odem MA, Eisenhoffer GT, Krachler AM (2020). The zebrafish as a model for gastrointestinal tract–microbe interactions. Cell Microbiol.

[14] Oehlers SH, Flores MV, Chen T, Hall CJ, Crosier KE, Crosier PS (2011). Topographical distribution of antimicrobial genes in the zebrafish intestine. Dev Comp Immunol.

[15] Willms RJ, Jones LO, Hocking JC, Foley E (2022). A cell atlas of microbe-responsive processes in the zebrafish intestine. Cell Rep.

[16] Massaquoi MS, Kong GL, Chilin-Fuentes D, Ngo JS, Horve PF, Melancon E (2023). Cell-type-specific responses to the microbiota across all tissues of the larval zebrafish. Cell Rep.

[17] Palma V, Gutiérrez MS, Vargas O, Parthasarathy R, Navarrete P (2022). Methods to evaluate bacterial motility and its role in bacterial–host interactions. Microorganisms.

[18] Wiles TJ, Schlomann BH, Wall ES, Betancourt R, Parthasarathy R, Guillemin K (2020). Swimming motility of a gut bacterial symbiont promotes resistance to intestinal expulsion and enhances inflammation. PLOS Biol.

[19] Holmberg A, Olsson C, Hennig GW (2007). TTX-sensitive and TTX-insensitive control of spontaneous gut motility in the developing zebrafish (Danio rerio) larvae. J Exp Biol.

[20] Rawls JF, Mahowald MA, Goodman AL, Trent CM, Gordon JI (2007). In vivo imaging and genetic analysis link bacterial motility and symbiosis in the zebrafish gut. Proc Natl Acad Sci.

[21] Wiles TJ, Jemielita M, Baker RP, Schlomann BH, Logan SL, Ganz J (2016). Host gut motility promotes competitive exclusion within a model intestinal microbiota. PLOS Biol.

[22] Jemielita M, Taormina MJ, Burns AR, Hampton JS, Rolig AS, Guillemin K (2014). Spatial and temporal features of the growth of a bacterial species colonizing the zebrafish gut. mBio.

[23] Sundarraman D, Smith TJ, Kast JVZ, Guillemin K, Parthasarathy R (2022). Disaggregation as an interaction mechanism among intestinal bacteria. Biophys J.

[24] Yang J, Shimogonya Y, Ishikawa T (2018). What causes the spatial heterogeneity of bacterial flora in the intestine of zebrafish larvae?. J Theor Biol.

[25] Molaei M, Barry M, Stocker R, Sheng J (2014). Failed escape: solid surfaces prevent tumbling of Escherichia coli. Phys Rev Lett.

[26] Berke AP, Turner L, Berg HC, Lauga E (2008). Hydrodynamic attraction of swimming microorganisms by surfaces. Phys Rev Lett.

[27] Li G, Tang JX (2009). Accumulation of microswimmers near a surface mediated by collision and rotational Brownian motion. Phys Rev Lett.

[28] Mok R, Dunkel J, Kantsler V (2019). Geometric control of bacterial surface accumulation. Phys Rev E.

[29] Yang J, Shimogonya Y, Ishikawa T (2019). Bacterial detachment from a wall with a bump line. Phys Rev E.

[30] Crawford RJ, Webb HK, Truong VK, Hasan J, Ivanova EP (2012). Surface topographical factors influencing bacterial attachment. Adv Colloid Interface Sci.

[31] Friedlander RS, Vlamakis H, Kim P, Khan M, Kolter R, Aizenberg J (2013). Bacterial flagella explore microscale hummocks and hollows to increase adhesion. Proc Natl Acad Sci.

[32] Feng G, Cheng Y, Wang S-Y, Borca-Tasciuc DA, Worobo RW, Moraru CI (2015). Bacterial attachment and biofilm formation on surfaces are reduced by small-diameter nanoscale pores: how small is small enough?. NPJ Biofilms Microbiomes.

[33] Taormina MJ, Hay EA, Parthasarathy R (2017). Passive and active microrheology of the intestinal fluid of the larval zebrafish. Biophys J.

[34] Rusconi R, Guasto JS, Stocker R (2014). Bacterial transport suppressed by fluid shear. Nat Phys.

[35] Kikuchi K, Noh H, Numayama-Tsuruta K, Ishikawa T (2020). Mechanical roles of anterograde and retrograde intestinal peristalses after feeding in a larval fish (Danio rerio). Am J Physiol-Gastrointest Liver Physiol.

[36] Kikuchi K, Haga T, Numayama-Tsuruta K, Ueno H, Ishikawa T (2017). Effect of fluid viscosity on the cilia-generated flow on a mouse tracheal lumen. Ann Biomed Eng.

[37] Okuyama K, Nishigami Y, Ohmura T, Ichikawa M. Accumulation of Tetrahymena pyriformis on Interfaces. Micromachines. 2021;12:1339. 10.3390/mi12111339 PMC8622496 34832750

