# Supplementary material for: Bacterial accumulation in intestinal folds induced by physical and biological factors
Source: BMC Biol. 2024 Apr 5;22:76. doi: 10.1186/s12915-024-01874-5 (PMC10998401; doi:10.1186/s12915-024-01874-5)
Supplement: Supplementary file 1 — Additional file 1: Movie S1. Geometry of the anterior intestinal lumen of zebrafish larvae in different sections of the z-axis. Movie S2. Bacterial swimming behavior in the larval zebrafish anterior intestine from the fold to the center. Movie S3. Bacterial swimming behavior in the larval zebrafish anterior intestinal fold. [file 12915_2024_1874_MOESM1_ESM.pptx]

## Slide 1
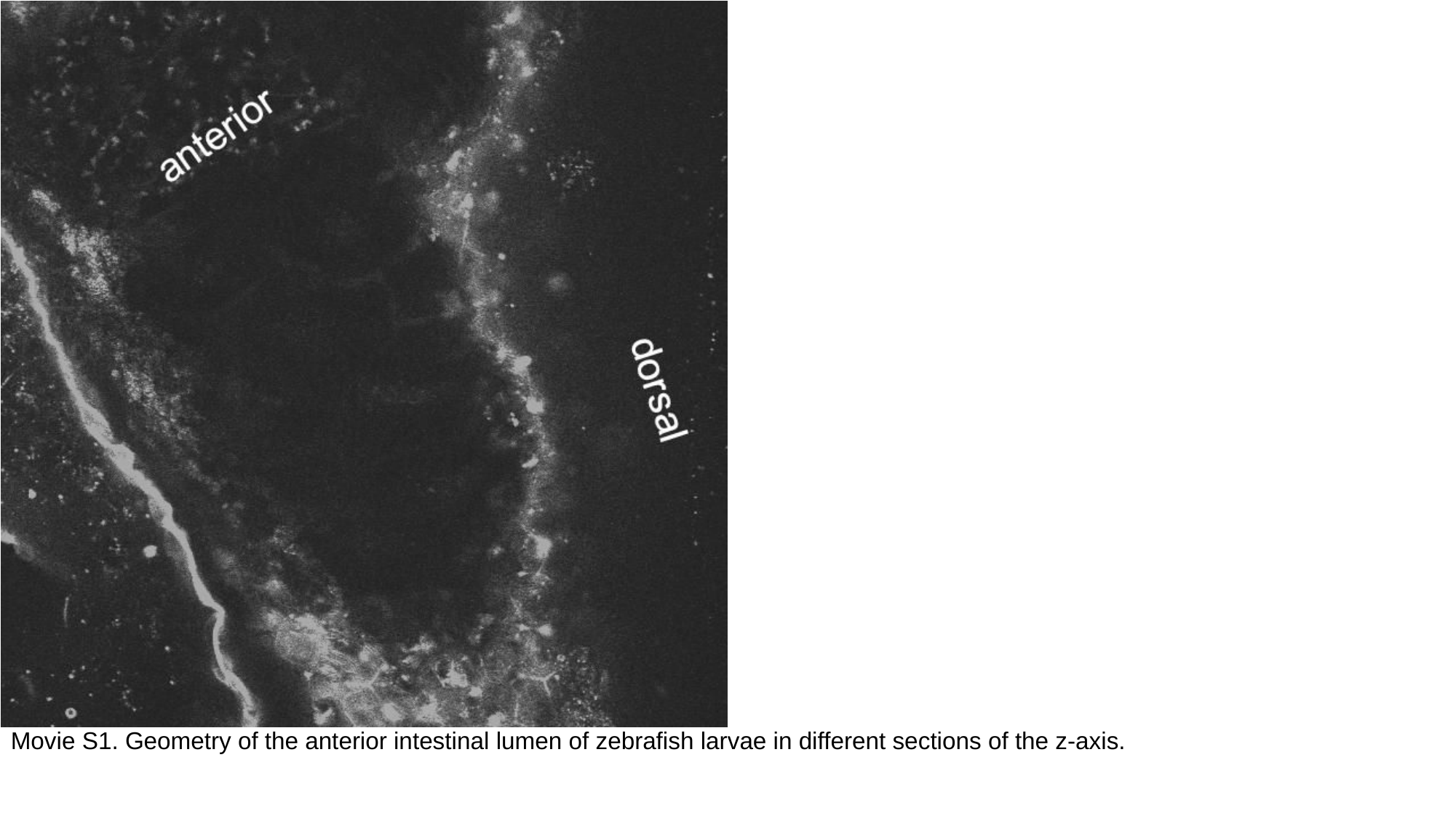

Movie S1. Geometry of the anterior intestinal lumen of zebrafish larvae in different sections of the z-axis.

## Slide 2
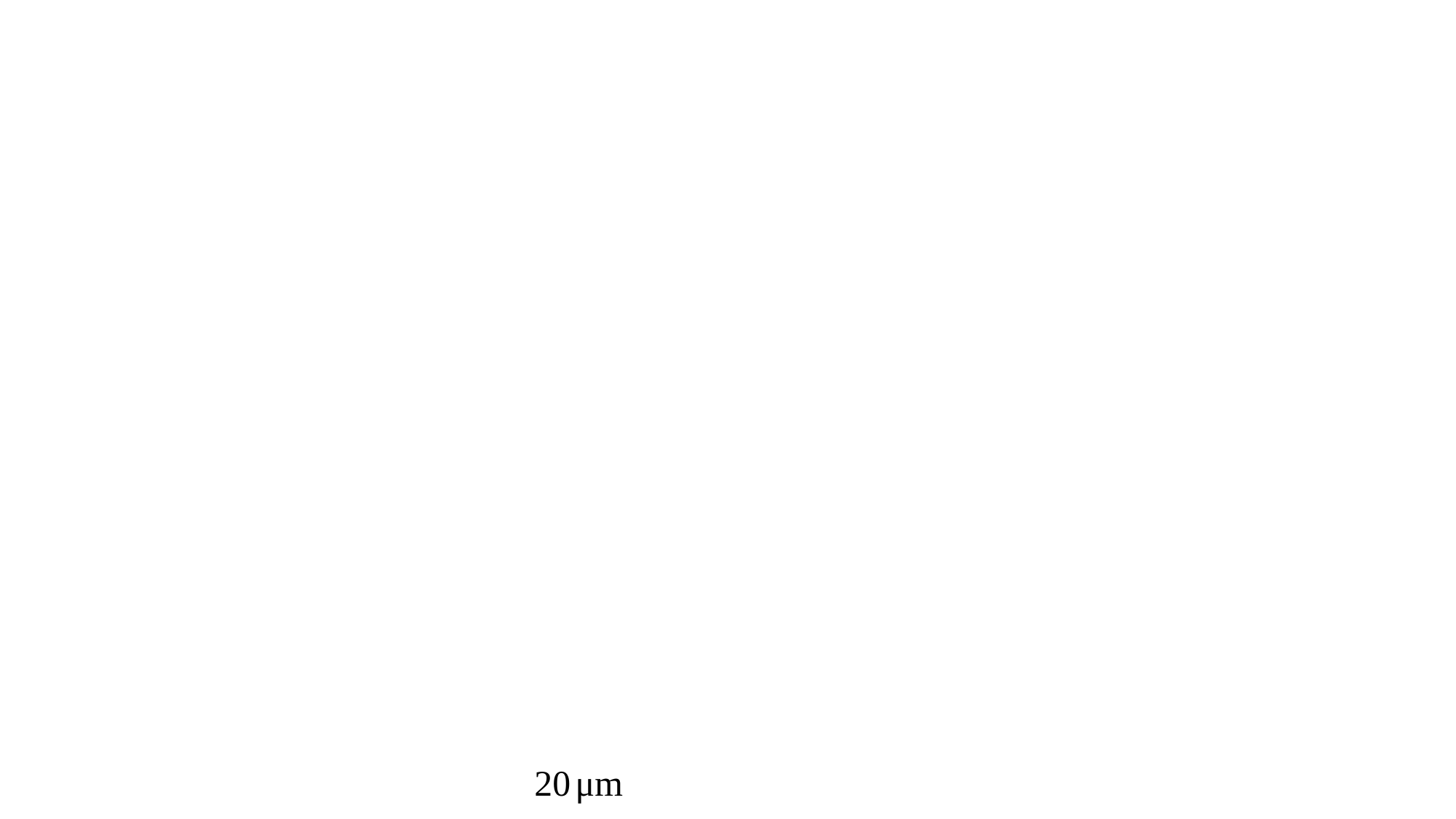

z
Dorsal
Anterior
Movie S2. Bacterial swimming behavior in the larval zebrafish anterior intestine from the fold to the center. 50 fps

## Slide 3
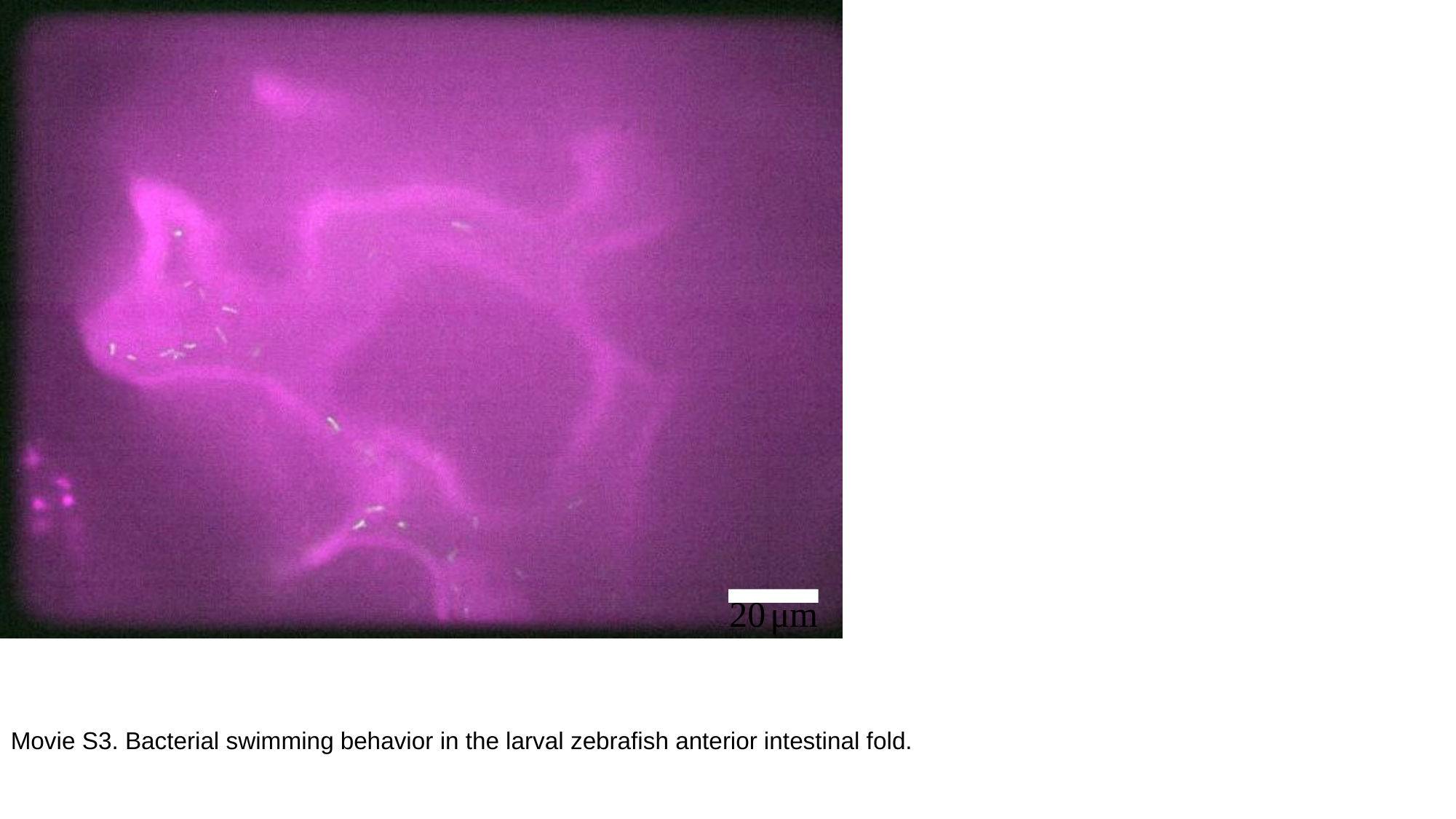

Movie S3. Bacterial swimming behavior in the larval zebrafish anterior intestinal fold.
