# Supplementary material for: Bacterial accumulation in intestinal folds induced by physical and biological factors
Source: BMC Biol. 2024 Apr 5;22:76. doi: 10.1186/s12915-024-01874-5 (PMC10998401; doi:10.1186/s12915-024-01874-5)
Supplement: Supplementary file 2 — Additional file 2: Figure S1. Trajectories of swimming bacteria in the confocal fluorescence microscope field of view of the middle section of the anterior intestinal lumen. Figure S2. Trajectories of swimming bacteria in the anterior intestinal folds. Figure S3. Areal number density of bacteria in different locations of the lumen of the middle intestine (near to the anterior intestine) at 0h. Figure S4. Areal number density and trajectories of fluorescent tracer particles in the anterior intestinal lumen. [file 12915_2024_1874_MOESM2_ESM.pdf]

Supplementary Figures

Bacterial accumulation in intestinal folds induced by physical and biological factors

Jinyou Yang[1*], Toma Isaka[2], Kenji Kikuchi[3,2], Keiko Numayama-Tsuruta[2], Takuji Ishikawa[2,3]

[1] School of Intelligent Medicine, China Medical University, Shenyang 110122, China
[2] Department of Biomedical Engineering, Graduate School of Biomedical Engineering, Tohoku University, 6-6-01 Aoba, Sendai 980-8579, Japan
[3] Department of Finemechanics, Graduate School of Engineering, Tohoku University, 6-6-01 Aoba, Sendai 980-8579, Japan

*Correspondence: jyyang@cmu.edu.cn

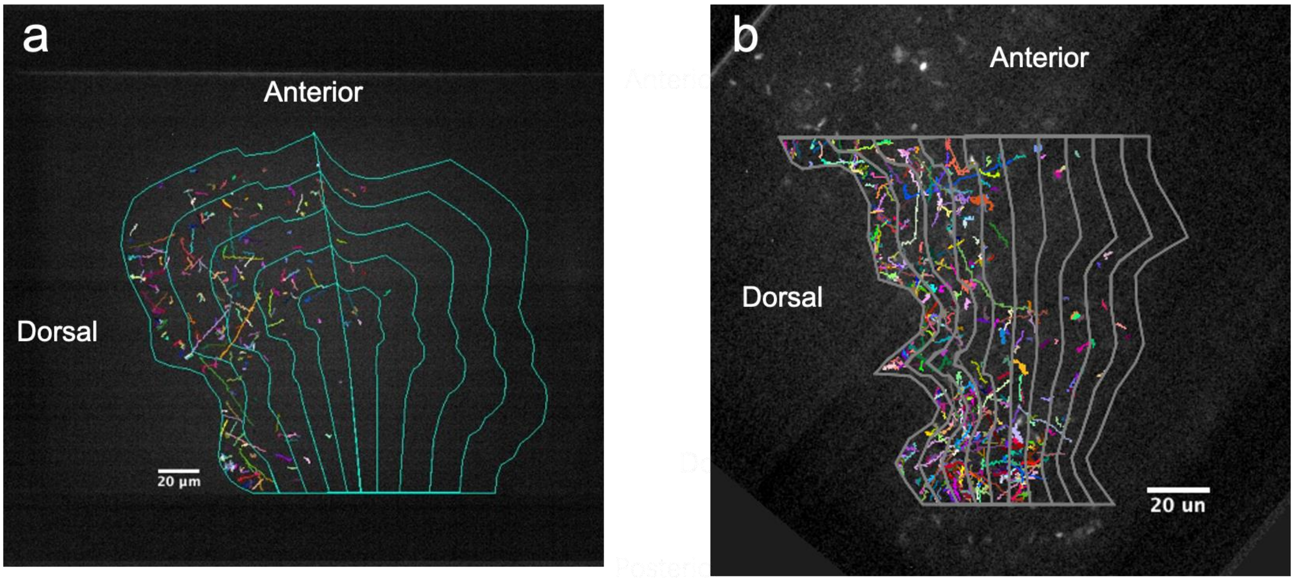

Figure S1. Trajectories of swimming bacteria in the confocal fluorescence microscope field of view of the middle section of the anterior intestinal lumen at 0 h (**a**) and 1 h (**b**) postinjection.

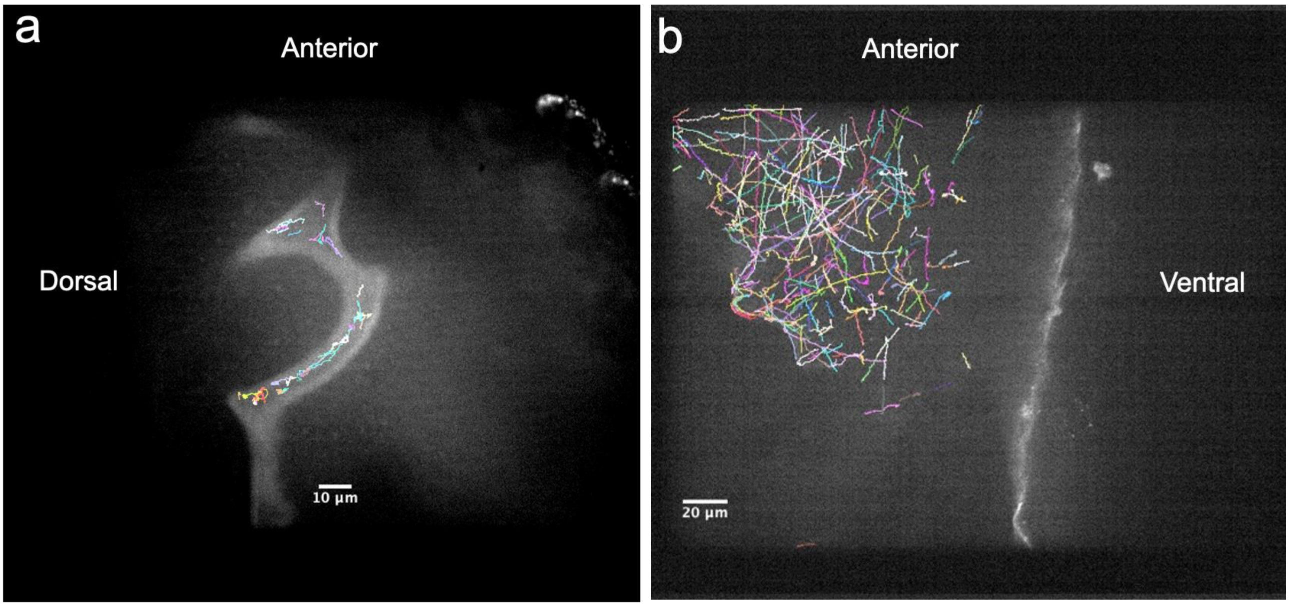

Figure S2. Trajectories of swimming bacteria in the anterior intestinal folds at 0 h (**a**) and 1 h (**b**) postinjection.

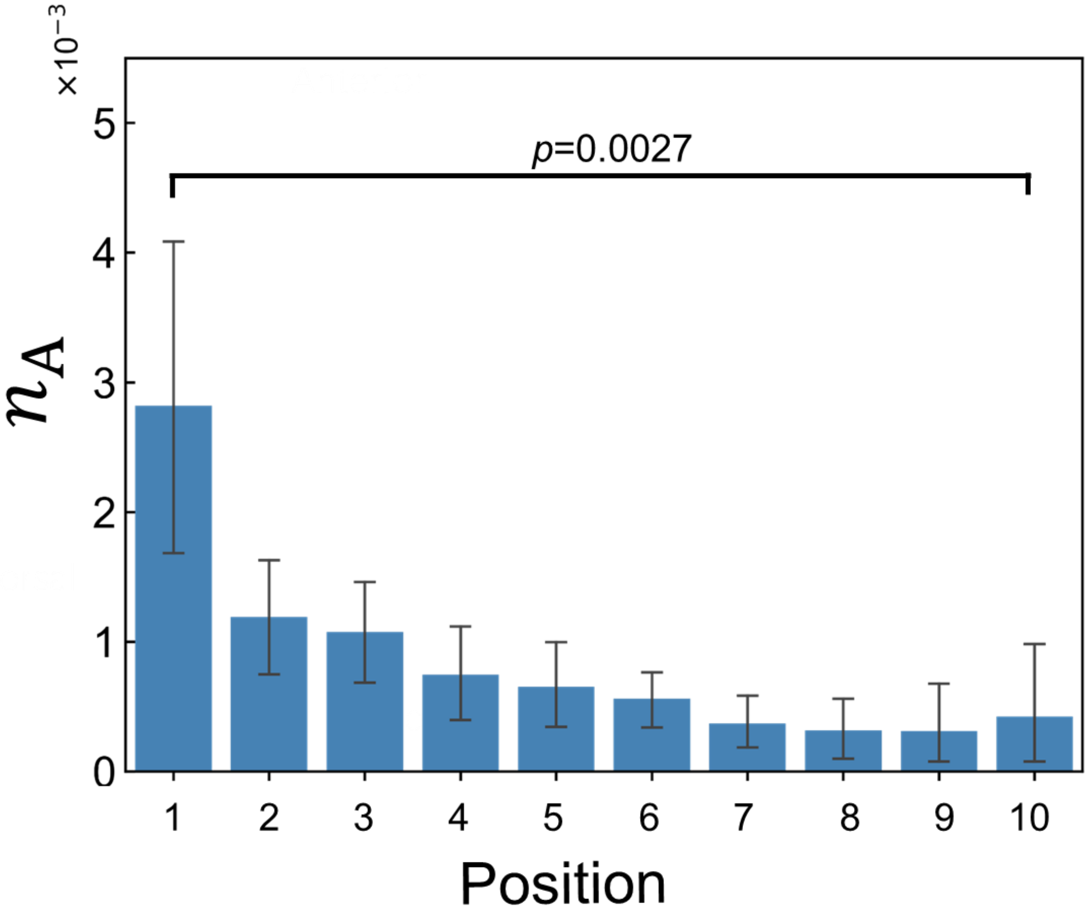

Figure S3. Areal number density of bacteria in different locations of the lumen of the middle intestine (near to the anterior intestine) at 0h. *p* value as determined by Mann-Whitney-Wilcoxon test, and error bars indicate standard deviation (N = 6 fish).

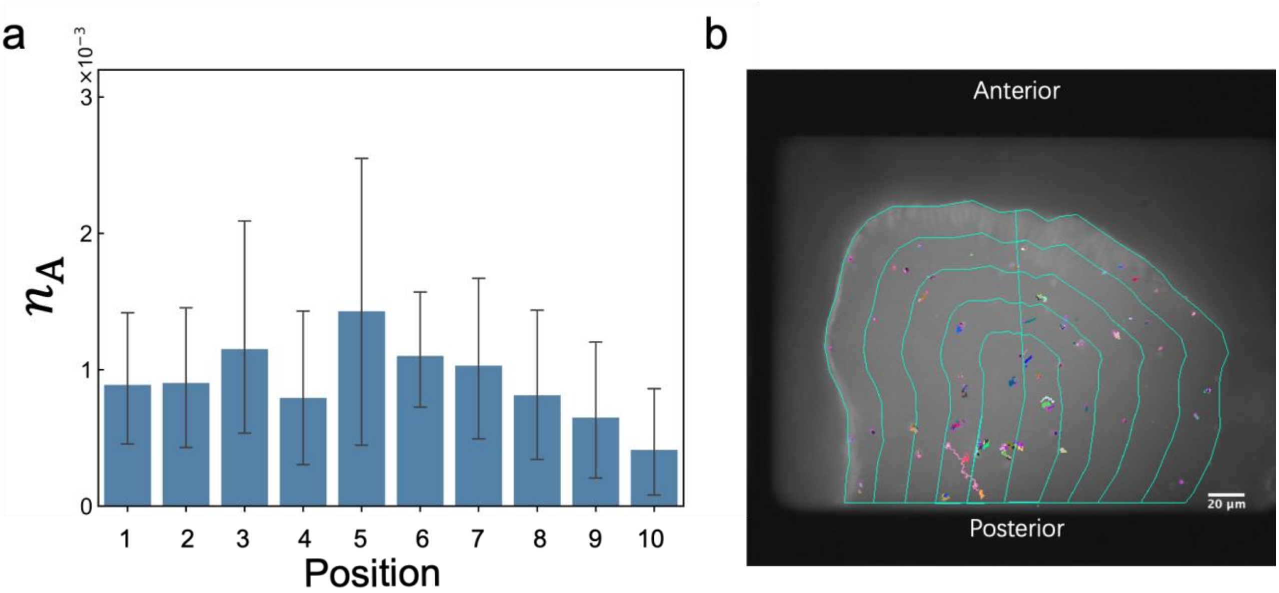

Figure S4. Areal number density and trajectories of fluorescent tracer particles in the anterior intestinal lumen. (**a**) Areal number density of fluorescent tracer particles in different locations of the lumen of the anterior intestine at 0 h, and error bars indicate standard deviation (N = 5 fish). (**b**) Trajectories of fluorescent tracer particles in the confocal fluorescence microscope field of view of the middle section of the anterior intestinal lumen at 0 h.
