# Supplementary material for: Bacterial accumulation in intestinal folds induced by physical and biological factors
Source: BMC Biol. 2024 Apr 5;22:76. doi: 10.1186/s12915-024-01874-5 (PMC10998401; doi:10.1186/s12915-024-01874-5)
Supplement: Supplementary file 3 — Additional file 3. The details of the continuum model. [file 12915_2024_1874_MOESM3_ESM.pdf]

Supplementary Theoretical analysis

Bacterial accumulation in intestinal folds induced by physical and biological factors

Jinyou Yang[1*], Toma Isaka[2], Kenji Kikuchi[3,2], Keiko Numayama-Tsuruta[2], Takuji Ishikawa[2,3]

[1] School of Intelligent Medicine, China Medical University, Shenyang 110122, China
[2] Department of Biomedical Engineering, Graduate School of Biomedical Engineering, Tohoku University, 6-6-01 Aoba, Sendai 980-8579, Japan
[3] Department of Finemechanics, Graduate School of Engineering, Tohoku University, 6-6-01 Aoba, Sendai 980-8579, Japan

*Correspondence: jyyang@cmu.edu.cn

1. Basic equations for the theoretical analysis

1.1 Normal diffusion flux

Consider a one-dimensional diffusion process of bacteria in the x-direction. Computational mesh is generated with interval dx as shown below. Let n_i [cells/m^3] be the number density of cells in mesh i, and v [m/s] is the swimming velocity of bacteria, which is assumed as constant in time and space.

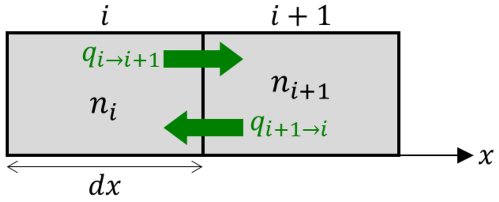

The cell flux $q_{i\to i+1}$ [cells/m^2 s] from mesh i to mesh $i+1$ as well as $q_{i+1\to i+1}$ from mesh $i+1$ to i can be given as follows

$$q_{i\to i+1} = n_i v \ ,$$
$$q_{i+1\to i} = -n_{i+1} v \ .$$

The net flux $q_{i,i+1}$ is

$$q_{i,i+1} = q_{i\to i+1} + q_{i+1\to i} = n_i v - n_{i+1} v \ .$$

The equation should be equivalent to Fick's law, i.e.

$$q_{i,i+1} = -D\frac{n_{i+1} - n_i}{dx} = -v(n_{i+1} - n_i) \ ,$$

where D is the diffusivity. By comparing these equations, we can derive the relation

$$v = \frac{D}{dx} \ .$$

1.2 Asymmetric flux near a wall

Let mesh 1 is in contact with a wall boundary and mesh 2 as shown below.

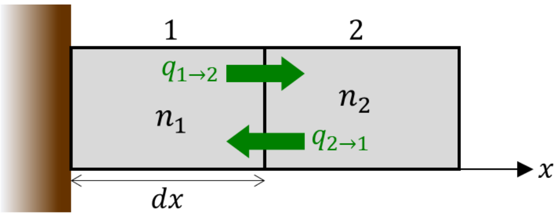

We assume that the flux $q_{2\to 1}$ is unchanged from the bulk condition and given as

$$q_{2\to1} = -n_2 v \ .$$

The flux $q_{1\to2}$ is reduced because cells prefer to stay near a wall. The reduced flux can be expressed as

$$q_{1\to2} = n_1 \alpha_w v \ ,$$

where α_w is a dimension-free parameter indicating the ratio of ensemble averaged bacterial velocity away from a wall to that toward the wall. The net flux is

$$q_{1,2} = q_{1\to2} + q_{2\to1} = n_1 \alpha_w v - n_2 v = -D\frac{n_2 - \alpha_w n_1}{dx} \ .$$

On the wall, there is no flux, so we have $q_{0,1} = 0$.

1.3 Flux due to taxis

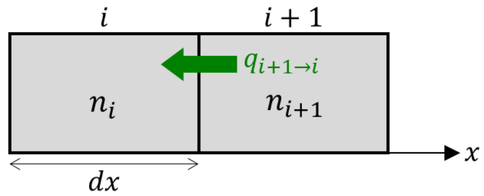

The flux induced by the directional movement, such as taxis, can be regarded as an advection flux. By introducing the advection velocity $-v_a$, the flux from mesh $i+1$ to i can be expressed as

$$q_{i+1\to i} = -n_{i+1} v_a \ .$$

We take the advection flux in the negative x direction, because the graph has larger peak in 1 region than 10 region.

1.4 Conservation of cells

In the bulk, the conservation of cells can be expressed using a control volume method as

$$\frac{\partial n_i}{\partial t} = v\frac{n_{i+1} - 2n_i + n_{i-1}}{dx} + v_a\frac{n_{i+1} - n_i}{dx}.$$

Péclet number Pe can be defined as the ratio of v_a to v, indicating the effect of the directional movement relative to the diffusion. Pe can also be expressed as

$$Pe = \frac{v_a}{v} = \frac{v_a dx}{D} \ .$$

We can nondimensionalize the equation by using dx as the characteristic length scale, and dx^2/D as the characteristic time scale. The equation can be transformed as

$$\frac{\partial n_i^*}{\partial t^*} = (1 + Pe)n_{i+1}^* - (2 + Pe)n_i^* + n_{i-1}^* \ ,$$

where $*$ indicates the dimensionless quantity. By using Euler explicit method for time-marching, we have

$$n_i^{*m+1} = n_i^{*m} + dt[(1 + Pe)n_{i+1}^* - (2 + Pe)n_i^* + n_{i-1}^*]^m \ ,$$

where dt is the time step, and m is the step number.

For mesh 1 next to the dorsal wall, we have the following equation

$$n_1^{*m+1} = n_1^{*m} + dt[(1 + Pe)n_2^* - \alpha_w n_1^*]^m \ .$$

For the last mesh M next to the ventral wall, we have the following equation

$$n_M^{*\,m+1} = n_M^{*\,m} + dt[-(\alpha_w + Pe)n_M^* + n_{M-1}^*]^m \ .$$

These equations are solved explicitly until the convergence is satisfied and the steady state solution is obtained.

1.5 Parameters

The width of zebrafish larval intestine is about 200 μm, and the body length of *E. coli* is about 2 μm. We set dx as 2 μm, and generate 100 mesh in the x direction. Thus, the characteristic length dx can be also regarded as the body length of *E. coli*.

The diffusivity of *E. coli* D in the bulk was measured as about 1.5 μm^2/s = 1.5×10^{-12} m^2/s (cf. Fig. 4e-f in the main text). The swimming speed of *E. coli* is about 20 μm/s, so the maximum value of $v_{a,max}$ is 20 μm/s. Using $dx = 2$ μm, we can derive the maximum value of Pe_{max} as

$$Pe_{max} = \frac{v_{a,max}}{v} = \frac{v_a dx}{D} = \frac{2 \times 10^{-5} \cdot 2 \times 10^{-6}}{1.5 \times 10^{-12}} = 13.3 \ \geq \ Pe \ .$$

α_w is the dimension-free parameter indicating the reduction ratio of wall-to-bulk velocity to the bulk velocity. Considering the wall accumulation of *E. coli*, α_w should satisfy $0 \leq \alpha_w \leq 1$.

The least square fitting of the numerical results to the experimental results indicates the most appropriate values for Pe and α_w to be $Pe = 0.018$ and $\alpha_w = 0.14$.
